# One and Five-Year Mortality Risk Prediction in Patients with Moderate and Severe Aortic Stenosis

**DOI:** 10.3390/jcm11102949

**Published:** 2022-05-23

**Authors:** Sameh Yousef, Andrea Amabile, Huang Huang, Ritu Agarwal, Saket Singh, Chirag Ram, Rita K. Milewski, Roland Assi, Yawie Zhang, Markus Krane, Arnar Geirsson, Prashanth Vallabhajosyula

**Affiliations:** 1 Division of Cardiac Surgery, Yale School of Medicine, New Haven, CT 06510, USA; sameh.yousef@yale.edu (S.Y.); andrea.amabile@yale.edu (A.A.); saket.singh@yale.edu (S.S.); chirag.ram@yale.edu (C.R.); rita.milewski@yale.edu (R.K.M.); roland.assi@yale.edu (R.A.); markus.krane@yale.edu (M.K.); arnar.geirsson@yale.edu (A.G.); 2 Section of Surgical Outcomes and Epidemiology, Yale School of Public Health, New Haven, CT 06510, USA; huang.huang@yale.edu (H.H.); yawie.zhang@yale.edu (Y.Z.); 3 Joint Data Analytics Team, Information Technology Service, Yale University, New Haven, CT 06520, USA; ritu.agarwal@yale.edu

**Keywords:** aortic, stenosis, mortality, risk, prediction

## Abstract

(1) Background: Our goal was to develop a risk prediction model for mortality in patients with moderate and severe aortic stenosis (AS). (2) Methods: All patients aged 40–95 years, with echocardiographic evidence of moderate and severe AS at a single institution, were studied over a median of 2.8 (1.5–4.8) years, between 2013–2018. Patient characteristics and mortality were compared using Chi-squares, t-tests, and Kaplan–Meier (KM) curves, as appropriate. The risk calculation for mortality was derived using the Cox proportional hazards model. A risk score was calculated for each parameter, and the total sum of scores predicted the individualized risks of 1-and 5-year mortality. (3) Results: A total of 1991 patients with severe and 2212 with moderate AS were included. Severe AS patients were older, had a lower ejection fraction %, were more likely to be Caucasian, and had lower rates of obesity and smoking, but had higher rates of cardiac comorbidities and AVR (49.3% vs. 2.8%, *p* < 0.0001). The unadjusted overall mortality was 41.7% vs. 41%, *p* = 0.6530, and was not different using KM curves (log rank, *p* = 0.0853). The models included only patients with complete follow-up (3966 in the 1-year, and 816 in the 5-year model) and included 13 variables related to patient characteristics, degree of AS, and AVR. The C-statistic was 0.75 and 0.72 for the 1-year and the 5-year models, respectively. (4) Conclusions: Patients with moderate and severe AS experience high morbidity and mortality. The usage of a risk prediction model may provide guidance for clinical decision making in complex patients.

## 1. Introduction

Calcific aortic stenosis (AS) is the most common valvular heart disease in Western countries, with at least one in every eight people suffering from moderate or severe disease [1,2]. AS is projected to increase in conjunction with the overall aging of the population and the persistent high burden of atherosclerotic risk factors [3]. The 2-year survival rate for severe symptomatic AS is less than 50%, worse than the mortality of many malignancies [4]. Moreover, patients with moderate AS experience high mortality according to recent reports [5,6,7,8].

AS primarily affects the elderly and shares similar biological and etiological risk factors with atherosclerotic diseases. Therefore, multiple comorbidities are usually prevalent in patients suffering from AS [9,10,11].

In such a complex patient population, the identification and integration of relevant prognostic information into the decision-making process is crucial. A better knowledge concerning prognostic outcome would allow for individualized patient encounters in the era of precision medicine. Yet, only few prognostic models have been developed for patients with AS. Most importantly, the current models have been limited to highly selected subgroups and are not widely applicable in practice: several reports focused on the mortality risk of medical management of AS classified by degree of stenosis, while others provided risk calculators for mortality after aortic valve replacement (AVR) [12,13,14].

Equally important is the fact that there is no proven medical therapy able to reverse or reduce the disease progression, and AVR continues to be the only effective treatment option that increases both survival and quality of life in patients with AS [15,16,17,18]. Still, a significant number of patients with severe AS do not undergo intervention [4,19,20].

Regarding this effect, we leveraged a large echocardiography database to develop a prediction model to better characterize risk factors of mortality, based on readily available information on demographics, comorbidity, and degree of stenosis. Furthermore, we aimed to analyze patient benefits form AVR intervention in the background of individualized risk profile stratification.

## 2. Materials and Methods

### 2.1. Data Source and Patient Population

The Institutional Review Board at Yale University approved this study. Yale New Haven Health System is a tertiary care center serving the community of the greater New Haven area, as well as a large portion of the population of the state of Connecticut (characterized by multiple ethnic backgrounds, age groups, and comorbidity profiles), in addition to the out-of-state referrals. All echocardiography data (both transthoracic and transesophageal) and electronic health records were queried for patients ≥18 years old who had at least one study during the calendar years of 2013 to 2018.

### 2.2. Analytic Cohort Building

Given that AS is rare before age 40, patients less than 40 years old at the time of their initial study were excluded. Patients older than 95 years old at the time of their initial study were also excluded because diagnosis of AS and AVR adds minimal benefit to longevity in such an old age group.

**Figure 1:**
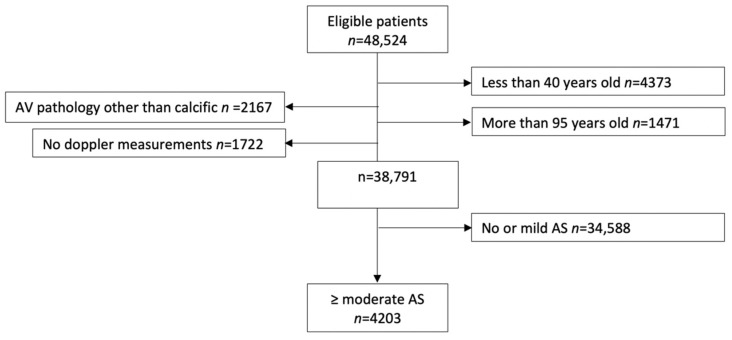
Cohort-building study flow diagram. Patients less than 40 and more than 95 years old, patients missing all echocardiographic parameters (AVA, DVI, V-max, PG-mean), and patients with valve pathology other than calcific (rheumatic, HOCM, tumor, endocarditis, and patients who had AVR as a part of aneurysm or dissection repair) were excluded.

Using the echocardiography reports and International Classification of Disease (ICD-10) codes, we excluded patients with a prosthetic aortic valve in their initial echocardiography during the study period, patients with AV pathology other than calcific AS (i.e., rheumatic AS, endocarditis, hypertrophic obstructive cardiomyopathy (HOCM), moderate and severe AI and aortic valve tumor), patients who had AVR as part of an aortic aneurysm or dissection repair, and patients who received heart transplantation or ventricular assist device treatment. Studies missing all AV doppler parameters were also excluded (Figure 1).

### 2.3. Aortic Stenosis Severity

Based on echocardiography parameters clinically used to define AS (namely aortic valve area (AVA), dimensionless valve index (DVI), mean pressure gradient across the valve (PG-mean), and the maximum flow velocity across the valve (V-max)), patients were categorized into: severe AS (AVA ≤ 1 cm^2^, or DVI ≤ 0.25, or V-max ≥ 4 m/s, or PG-mean ≥ 40 mmHg) and moderate AS (AVA 1–1.5 cm^2^, or DVI 0.25–0.5, or V-max 3–4 m/s, or PG-mean 20–40 mmHg) [21]. When multiple data were available, the most severe values were used to assess the degree of AS.

### 2.4. Patient Characteristics

Age was defined as the age at the time of the index echocardiography date. Race was categorized into Caucasian, African American, and other races. Baseline body surface area (BSA) was used. Smoking was defined by a positive history of more than 5 years of smoking. Comorbidities (hypertension, diabetes mellitus, dyslipidemia, heart failure, chronic kidney disease, coronary artery disease, chronic obstructive pulmonary disease, stroke, peripheral vascular disease, atrial fibrillation) were defined by ICD-10 codes (Appendix A).

### 2.5. Aortic Valve Replacement

Using unique identifiers (i.e., medical record number, last name, first name, and date of birth), patients with AS were linked to our institutional Society of Thoracic Surgeries (STS) database and the STS/ACC TVT TAVR registry.

### 2.6. Mortality Data

Death dates were extracted from the Connecticut State Vital Statistics database by linking the patient’s first and last name and date of birth on the date of censoring, 23 January 2020. The median follow-up time and inter-quartile range were 2.8 (1.52–4.8) years.

### 2.7. Statistical Analysis

Categorical variables were summarized as counts and percentages, while continuous variables were summarized as means and standard deviations (SD). Differences between the study groups (moderate vs. severe AS) were tested using the Student t-test for continuous variables and the Chi-square test for categorical variables. Mortality of the patients with moderate AS was compared to patients with severe AS using Kaplan–Meier (KM) curves.

Variables predicting risk of 1- and 5-year mortality were evaluated by Cox proportional hazards models in moderate and severe AS patients, with 1- and 5-year follow-up, respectively. A total of 237 patients who were followed-up for less than 1 year at the end of follow-up have been excluded from the 1-year mortality model, resulting in 3966 patients for the prediction analysis, while 3387 patients who were followed less than 5 years at the end of follow-up have been excluded from the 5-year mortality model, resulting in 816 patients. Candidate variables include information on demographics (age at diagnosis, sex, and race/ethnicity), comorbidities, AVR, and severity of AS.

Age was used as a continuous variable and as the age-squared term to further evaluate the nonlinear relationship between age and mortality. The covariates included in the final prediction model were selected by a combination of stepwise regression and Akaike information criteria [22]. The risk score of each parameter was calculated by dividing the corresponding β coefficient by the lowest β value and rounding to the closest integer. Risk scores of 1- and 5-year mortality for each individual patient were calculated as the sum of all parameter scores. Estimated risks of 1- and 5-year mortality were calculated based on the individual total risk score and average 1- and 5-year survival (S_t = 1 or 5_) [23]. Model performance was assessed using the C-index for discrimination ability and the visual estimation of the predictiveness curve for calibration.

The *p* value of <0.05 was defined a priori and was used to define statistically significant differences. Analysis was conducted using Microsoft Excel 2019 and Prism 8.2 (GraphPad Software, San Diego, CA, USA) for descriptive analyses and SAS 9.4 (SAS Institute Inc, Cary, NC, USA) and R 3.6.3 (R Foundation, Vienna, Austria) for survival analysis.

## 3. Results

Between 2013 and 2018, 48,524 patients ≥18 years old underwent at least one echocardiography study, with a total number of 132,116 studies available. A total of 38,791 patients were included in the final analysis after excluding patients <40 or >95 years old, patients with a prosthetic valve on their first study during the study period, patients with AV pathology other than degenerative (i.e., endocarditis, HOCM, moderate or higher aortic insufficiency) and studies with no single parameter available to assess AS severity (Figure 1). When stratified by degree of AS, 5.7% of them (*n* = 2212) had moderate AS, and 5.1% (*n* = 1991) had severe AS.

### 3.1. Patient Characteristics and Comorbidities

Table 1 summarizes patient characteristics: patients with severe AS were more likely to be older, Caucasian, with lower BSA, lower EF%, higher rates of cardiac comorbidities (CHD, Afib, and CHF), and were less likely to have a history of obesity and stroke. The rates of DM, dyslipidemia, CKD, COPD, PVD, and hypertension were not statistically different between moderate and severe AS patients.

Rates of AVR were different: 2.8% (*n* = 61) of moderate AS had AVR (all SAVR), while 46.8%(*n* = 931) with severe AS had AVR (TAVR = 63.5% and SAVR = 36.5%).

BSA: body surface area; CKD: chronic kidney disease; CHD: chronic heart disease; COPD: chronic obstructive pulmonary disease; PVD: peripheral vascular disease; AVA: aortic valve area; DVI: dimensionless valve index; LVIDD: left ventricular internal end diastolic diameter; LVISD: left ventricular internal end systolic diameter.

### 3.2. Comparative Mortality of Patients with Moderate and Severe AS

**Figure 2:**
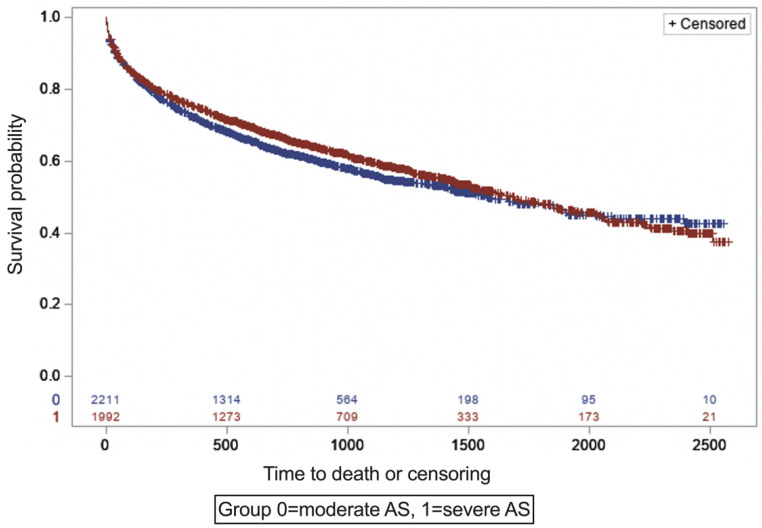
Mortality of patients with moderate and severe AS. Unadjusted mortality using Kaplan–Meier curves for 2 groups (moderate AS (blue curve) vs. severe AS (red curve)).

The unadjusted all-cause mortality rate throughout the study period was not statistically different in moderate vs. severe AS (moderate AS = 41%, and severe AS = 41.7%, *p* = 0.6530). The overall mortality rate in patients with moderate AS who had AVR was 31%, and in patients with moderate AS who did not undergo AVR, it was 41.3%. In patients with severe AS, the mortality rate in patients with AVR was 26% and in patients without AVR, it was 55%. In a time-to-event analysis using KM curves, patients with moderate AS had similar mortality rates compared to patients with severe AS (Figure 2).

### 3.3. Predicted Survival among Patients with Moderate and Severe AS

Variables selected as predictors for 1- and 5-year mortality and corresponding β coefficients are summarized in Table 2.

**Figure 3:**
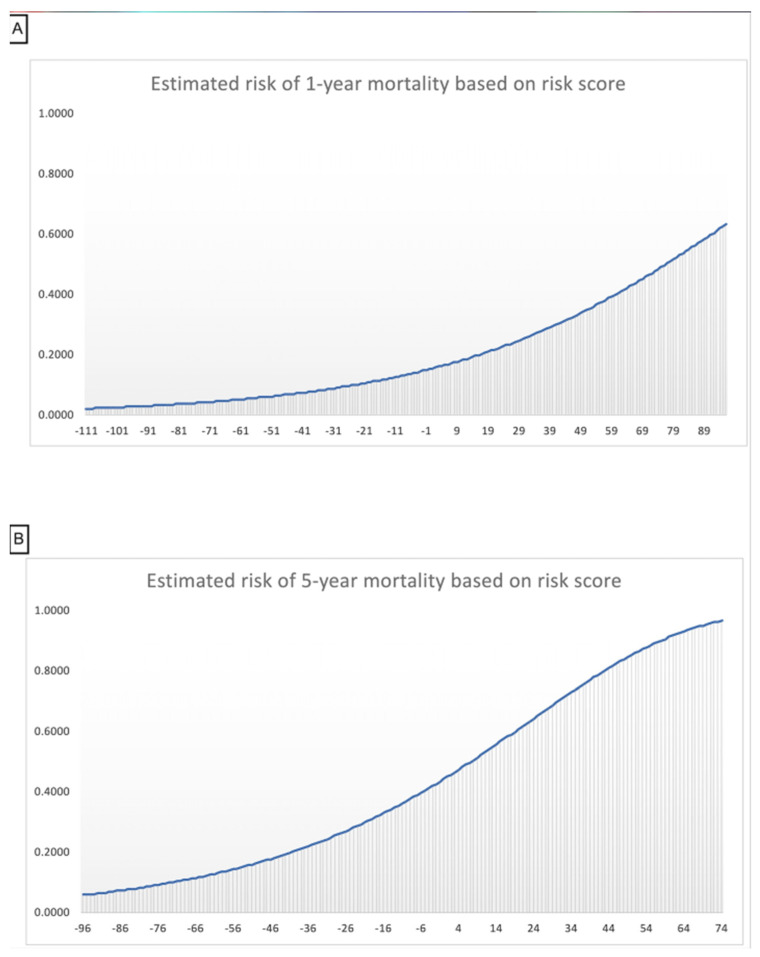
Risk of mortality based on risk scores. Panel (**A**): risk of 1−year mortality for risk scores. Panel (**B**): risk of 5−year mortality for risk scores.

Scores of risk factors (Table 3, Figure 3) indicated that AVR was the most significant negative predictor of both 1- and 5-year mortality. This was reflected in the β estimate (−1.3 for the 1-year and −1.2 for the 5-year model), the HR of mortality (0.27 CI [0.23–0.31] for the 1-year and 0.29 CI [0.23–0.37] for the 5-year model), and the integer score (−70 for the 1-year and −52 for the 5-year model). The degree of stenosis was a predictor of mortality in the 1-year model, where severe AS in reference to moderate AS was associated with increased risk of mortality (HR = 1.4 CI [1.3–1.6]), but not in the 5-year model (HR = 1.28 CI [0.98–1.5]). Age was associated with incremental increase in mortality; for each 10 year increase in age, the risk of mortality increased by 2%. There was no difference in mortality according to gender, while race other than Caucasian and African American was a negative predictor of mortality. Among the comorbidities, CKD, HF, DM, history of stroke, and COPD were positive predictors of mortality, while hypertension, dyslipidemia, and history of obesity were negative predictors. (Table 2).

Finally, the estimated cumulative risk of mortality was calculated as the sum of risk scores, and the cohort was further divided into risk groups according to the estimated risk of mortality (Table 4). The C-statistic was 0.75 and 0.72 for the 1-year and 5-year models, respectively. Visual assessment of the predictiveness curve supported good calibration (Appendix B).

## 4. Discussion

As the elderly population continues to expand, a better understanding of the mortality risk rendered by AS and associated comorbidities in this population is needed. Valvular intervention in this population has been shown to increase longevity and improve quality of life; therefore, AVR decisions need to be balanced with patient age, comorbidity burden, and quality of life. In our study, among the many studied parameters, the risk prediction model showed AVR to be the strongest negative predictor of mortality. Yet, a large fraction of patients did not undergo intervention. The low rate of AVR in patients with moderate AS (2.8% in this study) is understandable considering the current guidelines that recommend it as a class II-A indication for patients undergoing cardiac surgery for other indications [24], but the low rate of intervention in severe AS (43% in this study) underscores the gap that still exists in severe AS management. In reports before the era of TAVR, prohibitive surgical risk was the most common reason for lack of intervention [19,25,26]. The increasingly wider adoption of TAVR-enabled intervention in high surgical risk patients and its impact is reflected as a noted decline in national mortality from aortic stenosis [18]. In a more contemporary report in the TAVR era, 74% of patients with severe symptomatic AS who did not undergo intervention were not referred for evaluation [4], reflecting the need for increasing awareness and improved timely referral of AS patients.

To this effect, we report the development of a robust predictive model to help clinicians and patients make more informed decisions. At the point of care, it may be preferable to use individual survival probabilities calculated automatically by equations derived from the study model. These results would quantify risk as 1- and 5-year survival and thereby inform both the provider and the patient. Such an approach may incentivize provider recommendation of guideline-based care, as well as patient compliance.

For example, in a Caucasian male patient whose age is 78 and who has DM, CKD, and HF, and whose AS is severe, without AVR, this patient will have a sum of scores of 67 in the 1-year and 56 in the 5-year mortality models. This correlates with a 35–45% mortality risk within 1 year and a 85–95% mortality risk within 5 years. If the same patient undergoes AVR, the sum of scores will be -3 in the 1-year and 4 in the 5-year models. This correlates with a 10–20% mortality risk within the first year and a 38–52% mortality risk within 5 years. With these numbers available at the point of care, this patient will be able to understand the risks and make an informed decision.

In this cohort, patients with moderate AS were found to have high mortality approaching that of patients with severe AS; hence, they were included in the model. It is possible that even a moderate degree of AS could be detrimental in certain groups of patients. This is shown in our model as the sum of scores, highlighting the role for individualized prognostic models that include many patient factors, in addition to the degree of stenosis. These results are clinically supported by data from the National ECHO Database of Australia [6]. Moreover, on a pathological level, using speckle tracking MRI, Ng AC et al., showed successive impairment of multidirectional myocardial function with increasing severity of AS [27].

To facilitate effective use of the model, 13 variables—chosen by the step wise selection function of the Cox models—were included in the final model, and an integer score for each parameter based on the beta estimate of the variable importance in the model was calculated. Using the Cox model in this analysis enabled us to account for the time-sensitive nature of mortality in this patient population. However, we still reported the 1- and 5-year risks to make it easier for patients to comprehend the risk of mortality in an objective numerical way during point-of-care conversations.

### Limitations

This is an observational retrospective study that explores the prognostic significance of certain variables in patients with advanced AS. In this framework, causality cannot be established, and the interpretation is limited to a possible association of certain variables with mortality in this patient population. Further studies, preferably randomized control studies, are suggested to explore the impact of AVR in patients with moderate AS. The low rate of intervention in this study could be explained by the fact that TAVR was not fully employed during the early years of the study. However, contemporary reports show continued under-utilization of this resource, even in academic centers [28]. The prediction model presented in this study is based on claims data in the form of ICD10 codes, which limited our ability to include important factors in the model. For example, it not possible to extract data on functional status and frailty index from these claims data. Future work should consider these factors in the prognostic models.

## 5. Conclusions

In this study of echocardiograms obtained across a large health system encompassing various care settings, patients with severe, as well as moderate, AS experienced high morbidity and mortality. The prognostic model developed in this study has the potential to be used as an individualized mortality risk calculator during points-of-care.

## Figures and Tables

**Table 1:** Characteristics of patients with AS on their first echocardiography during the study period *.

	Moderate AS *n* = 2212	Severe AS *n* = 1991	*p*
Age/year	76.09 ± 11.5	80.26 ± 10.6	<0.0001
Sex (male)	1178 (53.3%)	1043 (52.4%)	0.5730
Race—Caucasian	1843 (83.3%)	1764 (88.6%)	<0.0001
Race—African American	206 (9.3%)	119 (6%)	
Race—Other	156 (7.1%)	108 (5.4%)	
BSA (m^2^)	1.89 ± 0.28	1.86 ± 0.28	<0.0001
Smoking	694 (31.4%)	560 (28.1%)	0.0216
Ejection fraction (%)	57.01 ± 14	56.68 ± 14.62	<0.0001
Diabetes mellitus	726 (32.8%)	615 (30.9%)	0.1797
Dyslipidemia	1285 (58.1%)	1194 (60%)	0.2166
Obesity	181 (8.2%)	121 (6.1%)	0.0083
CKD	345 (15.6%)	328 (16.5%)	0.4387
Stroke	303 (13.7%)	226 (11.4%)	0.0220
Atrial fibrillation	555 (25.1%)	602 (30.2%)	0.0002
CHD	751 (34%)	834 (41.9%)	<0.0001
COPD	359(16.2%)	323 (16.2%)	0.9953
PVD	222 (10%)	230 (11.6%)	0.1132
Heart failure	409 (18.5%)	453 (22.8%)	0.0006
Hypertension	1708 (77.2%)	1560 (78.4%)	0.3760
AV intervention	61 (2.8%)	931 (46.8%)	<0.0001
Peak Velocity (m/sec)	2.82 ± 0.65	4.1 ± 0.34	<0.0001
Mean Gradient (mm Hg)	18.7 ± 8.2	41 ± 5.6	<0.0001
AVA (cm^2^)	1.4 ± 0.28	0.78 ± 0.22	<0.0001
DVI	0.46 ± 0.13	0.25 ± 0.08	<0.0001
LVIDD (cm)	4.72 ± 0.81	4.63 ± 0.79	<0.0001
LVISD (cm)	3.4 ± 0.58	3.2 ± 0.88	0.0004
Overall mortality	907 (41%)	830 (41.7%)	0.6530

* Continuous variables are represented as mean ± SD, and categorical variables are represented as number (%). Comparisons of continuous variables by Student *t*-test and categorical variables by Chi square test.

**Table 2:** Cox proportional regression coefficients and hazard ratios for AS patients with 1-year and 5-year follow-up.

Risk Factor	Patients with 1-Year Follow-up					Patients with 5-Year Follow-up				
	Number	β Coefficient	*p*-Value	Hazard Ratio	95% CI	Number	β Coefficient	*p*-Value	Hazard Ratio	95% CI
Age *	3966	0.0188	<0.0001	1.02	1.016–1.022	816	0.0237	<0.0001	1.02	1.018–1.030
Gender										
Female	1866	ref.		1.00						
Male	2100	0.0792	0.11	1.08	0.98–1.19					
Race/ethnicity										
Caucasian	3418	ref.		1.00		713	ref.		1.00	
African American	296	−0.0788	0.41	0.92	0.77–1.11	51	−0.0053	0.98	1.00	0.69–1.43
Another race	252	−0.3039	0.0071	0.74	0.59–0.92	52	−0.4021	0.040	0.67	0.46–0.98
Comorbidity										
Diabetes mellitus										
No	2711	ref.		1.00		555	ref.		1.00	
Yes	1255	0.1690	0.0018	1.18	1.07–1.32	261	0.3677	0.0002	1.44	1.19–1.75
Dyslipidemia										
No	1642	ref.		1.00		335	ref.		1.00	
Yes	2324	−0.1965	0.0001	0.82	0.74–0.91	481	−0.2542	0.0092	0.78	0.64–0.94
Obesity										
No	3692	ref.		1.00						
Yes	274	−0.1599	0.14	0.85	0.69–1.05					
CKD										
No	3340	ref.		1.00		693	ref.		1.00	
Yes	626	0.3828	<0.0001	1.47	1.30–1.66	123	0.4721	<0.0001	1.60	1.28–2.01
Stroke										
No	3462	ref.		1.00		696	ref.		1.00	
Yes	504	0.1899	0.0055	1.21	1.06–1.38	120	0.2202	0.061	1.25	0.99–1.57
COPD										
No	3317	ref.		1.00		654	ref.		1.00	
Yes	649	0.3161	<0.0001	1.37	1.22–1.55	162	0.1632	0.13	1.18	0.95–1.46
Heart failure										
No	3144	ref.		1.00		570	ref.		1.00	
Yes	822	0.2129	0.0002	1.24	1.11–1.38	246	0.2031	0.036	1.23	1.01–1.48
Hypertension										
No	892	ref.		1.00		183	ref.		1.00	
Yes	3074	−0.1066	0.087	0.90	0.80–1.02	633	−0.3824	0.0007	0.68	0.55–0.85
AS Severity										
Moderate	2101	ref.		1.00		368	ref.		1.00	
Severe	1865	0.3721	<0.0001	1.45	1.30–1.61	448	0.1889	0.076	1.21	0.98–1.49
Intervention										
No	2985	ref.		1.00		476	ref.		1.00	
Yes	981	−1.3131	<0.0001	0.27	0.23–0.31	340	−1.2336	<0.0001	0.29	0.23–0.37

*: Age was squared for each 10-year increment from 40. CKD: chronic kidney disease; CODP: chronic obstructive pulmonary disease.

**Table 3:** Score system for AS patients with 1-year and 5-year follow-up.

Risk Factor	Patients with 1-Year Follow-up		Patients with 5-Year Follow-up	
	β Coefficient	Score	β Coefficient	Score
Age	0.0188		0.0237	
40–49		0		0
50–59		1		1
60–69		2		2
70–79		3		3
80–89		4		4
90–99		5		5
Gender				
Female	ref.	0		
Male	0.0792	4		
Race/ethnicity				
Caucasian	ref.	0	ref.	0
African American	−0.0788	−4	−0.0053	0
Another race	−0.3039	−16	−0.4021	−17
Comorbidity				
Diabetes mellitus				
No	ref.	0	ref.	0
Yes	0.1690	9	0.3677	16
Dyslipidemia				
No	ref.	0	ref.	0
Yes	−0.1965	−10	−0.2542	−11
Obesity				
No	ref.	0		
Yes	−0.1599	−9		
CKD				
No	ref.	0	ref.	0
Yes	0.3828	20	0.4721	20
Stroke				
No	ref.	0	ref.	0
Yes	0.1899	10	0.2202	9
COPD				
No	ref.	0	ref.	0
Yes	0.3161	17	0.1632	7
Heart failure				
No	ref.	0	ref.	0
Yes	0.2129	11	0.2031	9
Hypertension				
No	ref.	0	ref.	0
Yes	−0.1066	−6	−0.3824	−16
AS Severity				
Moderate	ref.	0	ref.	0
Severe	0.3721	20	0.1889	8
Intervention				
No	ref.	0	ref.	0
Yes	−1.3131	−70	−1.2336	−52

**Table 4:** Risk estimates of 1- and 5-year mortality for AS patients with 1-year and 5-year follow-up *.

Patients with 1-Year Follow-up		Patients with 5-Year Follow-up	
Total Score	Estimate Risk of 1-Year Mortality	Total Score	Estimate Risk of 5-Year Mortality
−111 to −90	0.0201–0.0296	−96 to −90	0.0582–0.0668
−89 to −70	0.0302–0.0429	−89 to −70	0.0683–0.1050
−69 to −50	0.0437–0.0618	−69 to −50	0.1074–0.1633
−49 to −30	0.0630–0.0888	−49 to −30	0.1669–0.2490
−29 to −10	0.0904–0.1266	−29 to −10	0.2542–0.3688
−9 to 10	0.1289–0.1790	−9 to 10	0.3757–0.5224
11 to 30	0.1820–0.2496	11 to 30	0.5308–0.6949
31 to 50	0.2537–0.3418	31 to 50	0.7035–0.8515
51 to 70	0.3470–0.4562	51 to 70	0.8581–0.9533
71 to 96	0.4625–0.6296	71 to 74	0.9566–0.9656

* Average 1-year survival = 0.7310; 5-year survival = 0.4154.

## Data Availability

Not applicable.

## Appendix A

**Table A1:** **T****able****A1.** ICD-10 codes used to define comorbidities.

Disease	ICD10 Code
Aortic insufficiency	I35.1
Atrial fibrillation	I48.91, I48.0
Coronary artery disease	I25.10, I21.9, I25.5, I25.2
Stroke	I61.9, I63.9
Chronic kidney disease	N18.6, N18.9, N18.3, N18.6, Z99.2
Chronic obstructive pulmonary disease	J44.9
Endocarditis	I38
Heart failure	I50.9
Heart transplant status	Z94.1
Ventricular assisted device	Z95.811, Z95.812
Hyperlipidemia	E78.5, E78.00
Hypertrophic obstructive cardiomyopathy	I42.1, I42.2
Hypertension	I10
Obesity	E66.9, E66.01
Prosthetic heart valve	Z95.2, Z95.1
Peripheral vascular disease	I73.9
Diabetes mellitus	E10.9, E11.9, E11.65

ICD-10: international classification of disease codes, version 10.

## Appendix B

Predictiveness curves for 1- and 5-year mortality models.
